# Expression analysis of box C/D snoRNAs with SNPs between C57BL/6 and MSM/Ms strains in male mouse

**DOI:** 10.1371/journal.pone.0288362

**Published:** 2023-07-10

**Authors:** Rumiko Saito, Maina Otsu, Hidenori Kiyosawa, Gota Kawai

**Affiliations:** 1 Department of Life Science, Faculty of Advanced Engineering, Chiba Institute of Technology, Narashino, Chiba, Japan; 2 Department of Environmental Medicine, Kochi Medical School, Kochi University, Oko-cho, Nankoku, Kochi, Japan; University of Crete & IMBB-FORTH, GREECE

## Abstract

MSM/Ms mouse derived from the Japanese wild mouse has unique characteristics compared to the widely used C57BL/6 mouse. To examine the usefulness of the MSM/Ms mouse for the comparative genomic analysis, expression of small RNAs were analyzed by the large-scale sequence analysis for two strains of mouse, C57BL/6 and MSM/Ms. As a trial, expression of box C/D snoRNAs, which are the most abundant small RNAs in the cell, were analyzed. By the comparison of the read number for each fragment, 11 snoRNAs with single nucleotide polymorphisms (SNPs) were detected. One of the snoRNAs, SNORD53, shows the expression only for MSM/Ms and this snoRNA has a mutation in the box sequence in C57BL/6. Thus, it was demonstrated that the proposed experimental system using SNPs can give new insight for the gene expression regulation.

## Introduction

Among the experimental mouse strains (*Mus musculus*), C57BL/6 mouse (B6) has been one of the most popular strains used in a variety of research fields [1–3]. Although more than 20 substrains have been established for B6, it has been widely used as a background strain [1–3]. In contrast, MSM/Ms or Mishima mouse (MSM) shows unique characteristics compared to B6 [4–6]. For example, MSM is less likely to develop tumors than B6 [7], and MSM has tolerance to deafness [8], or behavioral traits such as active athletic activity and aggression [9, 10]. The genome sequence of MSM differs by about 1% compared to B6 [11, 12]. Thus, comparative genomics with B6 and MSM should contribute to deeper understanding of the gene regulation in mouse.

It is well known that many small-sized RNAs were expressed with important biological roles in the cell. Recently, small RNAs (about 60 to 80 nt) were found during an analysis of transcription from the sense–antisense transcripts (SAT) loci [13, 14]. The small RNAs were detected from the exon-overlapping regions of SAT loci and these RNAs may have regulatory roles in the mammalian genome. Thus, the search for small RNAs is expected to yield novel biological function.

In the present study, we focused on the expression of small nucleolar RNAs (snoRNAs) as a trial expression analysis because these are the most abundant small RNAs in cells. There are two types of snoRNA, C/D box snoRNA (SNORD) and H/ACA box snoRNA (SNORA), where SNORD forms a loose stem-loop and SNORA forms a tight two-hairpin structure [15]. Many snoRNAs guide the site-specific chemical modification of ribosomal RNA [16] and some snoRNAs are located at the imprinted loci [17]. The comparison of snoRNA expressions is anticipated to give novel insights into the mechanism of genomic imprinting.

To analyze small RNAs, we performed the next generation sequencing (NGS) analysis of small RNA fractions to find several novel structured small RNAs from the brain of B6 [18]. In the present study, we applied similar technique to B6 and MSM toward the comparative genomics of small non-coding RNAs. As the first trial, we focused on snoRNAs which are abundant and have several important functions in the cell as described above. This method can be used for analysis of differential expression of several snoRNAs.

## Materials and methods

### Preparation of RNA samples

RNAs were extracted from male mouse (C57BL/6J, male, 8–10 weeks, CLEA, Tokyo, Japan) and MSM/Ms (male, 8–10 weeks, provided by RIKEN BRC) brain by using TRIzol reagent (Invitrogen) according to the manufacturer’s protocol. Brains from typically two mice were homogenized for each subspecies before RNA extractions. Total RNA (60 μg) was then separated by 12% polyacrylamide/7M urea denaturing gel electrophoresis. The gel was stained with ethidium bromide and irradiated with UV light to detect RNA bands. Small RNAs of about 40 to 140 nt in length were recovered from the gel to obtain 2.2 and 3.2 μg/μl samples for B6 and MSM, respectively. Mouse experiments were performed in accordance with the institutional guidelines of Chiba Institute of Technology and Kochi University, and approved by the Animal Care and Use committees of both institutions under the approval numbers of 18002 and J-00022, respectively. The mice were properly sacrificed by cervical dislocation.

### Sequencing

DNA libraries for sequencing were prepared with three kinds of preparation kits; TruSeq Small RNA Library Prep Kit (TruSeq, Illumina, Inc.) [19], NEBNext Multiplex Small RNA Library Prep Set for Illumina (NEBNext, New England BioLabs Inc.) and SMARTer® microRNA-Seq Kit (SMARTer, Takara Bio USA, Inc.) [20–22]. In general, manufacturer’s protocols were used except for the final size selection step. In the final step, DNAs corresponding to small RNAs were selected by the SPRIselect size selection kit (Beckman Coulter, Inc.) with the double side selection protocol. The qualities of the DNA libraries were confirmed by the Bioanalyzer 2100 (Agilent Technologies) with the High Sensitivity DNA kit. The concentrations of the DNA libraries were around 5 nM. The sequence analyses for 151 nt in paired end were done by the MiniSeq system (Illumina) with the MiniSeq High Output Kit (300 Cycles) and the RNA sequences were extracted by home-made programs as described in Kiyosawa et al. 2015; for each set of 150 nt pair-ended read, sequences with overlapping at least 10 nt are extracted as a small RNA.

### Data analysis

The obtained RNA sequences in length of 10–292 were processed by mainly Microsoft Excel and home-made programs in part. Sequences for mouse brains of B6 and MSM were compared to each other and the sequences with more than 100-fold change between MSM and B6 or more than 100 for one strain and 0 for the other strain in read number per million total read number (rpm) were selected. Each sequence was mapped on the mouse genome (GRCm39/mm39) using BLAT in UCSC Genome Browser (https://genome.ucsc.edu) or subjected to the BLAST search in the snoRNA orthological gene database snOPY [23].

## Results

### Detection of differential expressed sequences

**Fig 1:**
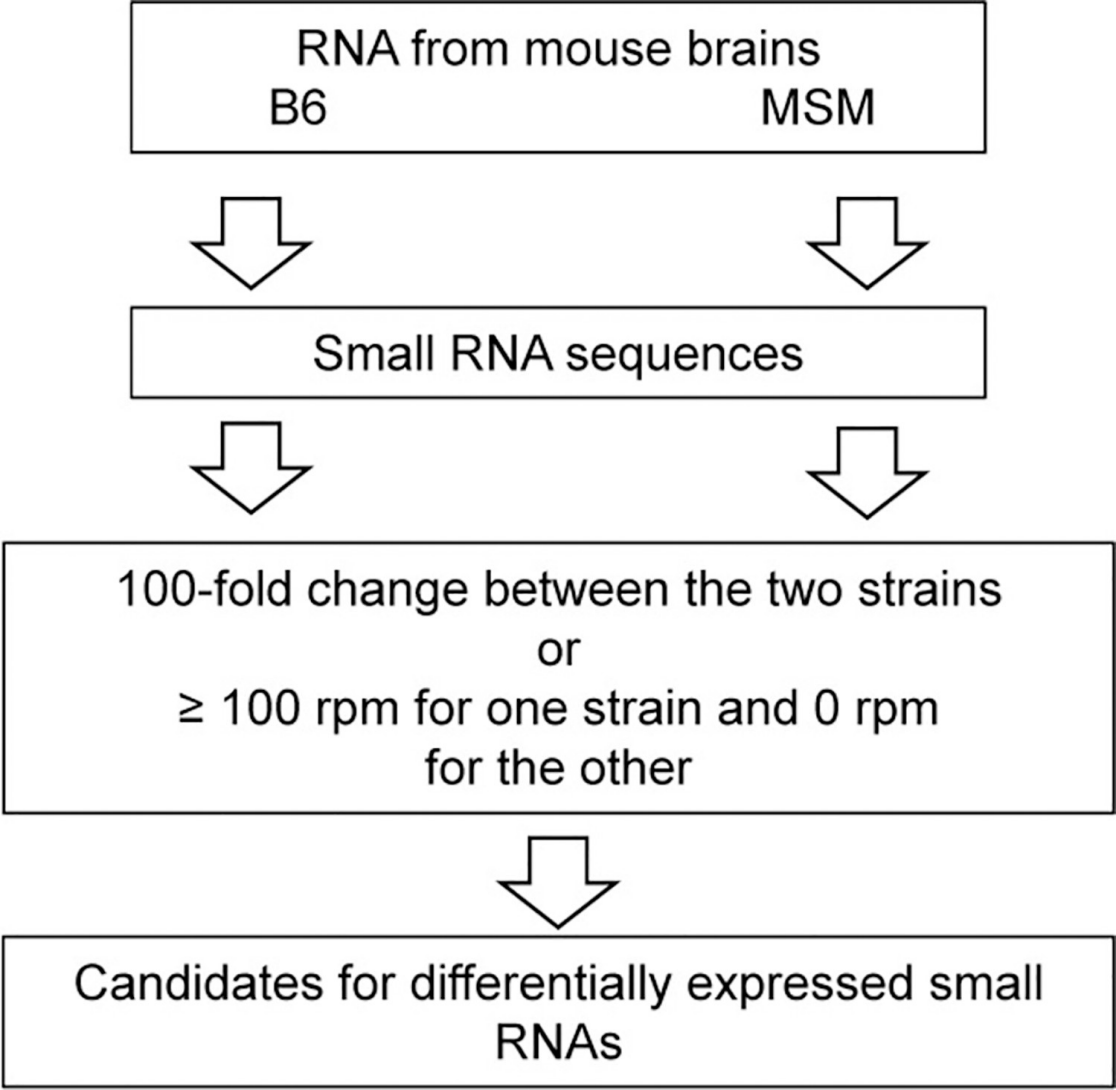
Scheme of the analysis performed in this study. rpm: read number per million total read number.

**Fig 2:**
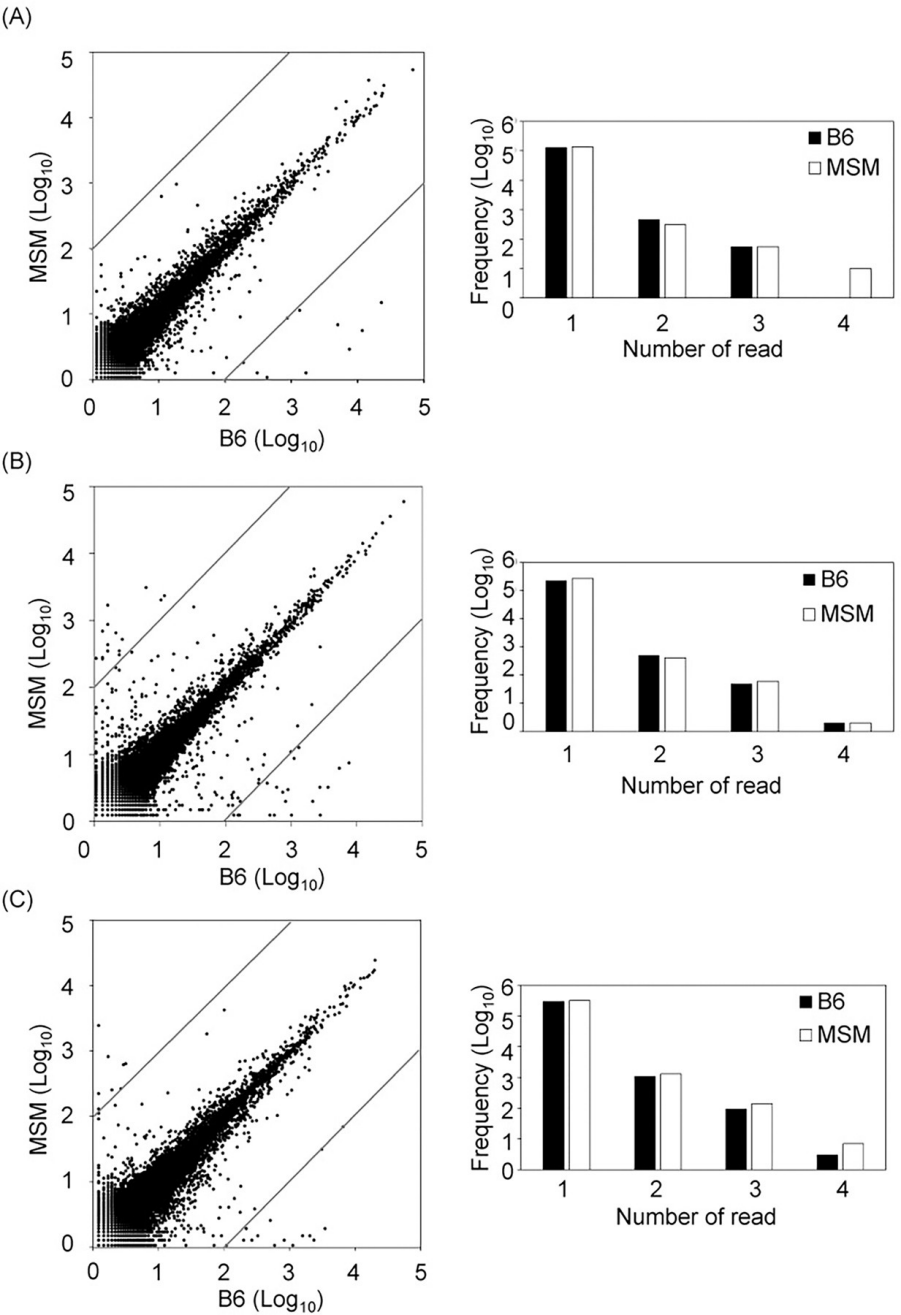
Comparison of small RNAs in B6 and MSM. (A-C) are corresponding to the analysis 1, 2 and 3, respectively. Left: correlation in number of reads for each fragment (rpm) between B6 and MSM. Lines indicate the 100-fold difference. Right: distribution of read numbers for fragments which were detected only one strain. The categories 1–4 indicate x < 10, 10 ≤ x < 100, 100 ≤ x < 1,000 and 1,000 ≤ x (rpm), respectively.

The overview of the analysis in this work was summarized in Fig 1. RNAs were extracted from brain of B6 and MSM, and the DNA libraries were prepared with three kinds of DNA library preparation kits, TruSeq, NEBNext and SMARTer. These kits are optimized for small RNAs such as miRNAs. Then, the sequences for each DNA libraries were analyzed and RNA sequences were extracted from the obtained sequence reads. Hereafter, the three data obtained from the three libraries were called as analyses 1–3, respectively. By an NGS analysis, 30,063,505 reads were obtained in total and 27,261,887 RNA sequences with length from 10 to 292 which cover the most of SNORD and SNORA. Hereafter, the number of RNA sequences are called as read number. As shown in Table 1, RNA sequences of 3,767,220–5,498,823 were obtained for each DNA library which contained RNA fragments of 180,679–406,614. To find candidates for the differentially expressed RNAs, correlation of the read numbers for each sequence were analyzed as shown in Fig 2. As shown in Fig 1, RNAs with 100-fold differences in rpm were selected. In each analysis, most of RNAs shows similar read numbers between B6 and MSM (Fig 2, left for each panel) and small number of RNAs showed 100-fold differences (outside of the lines in Fig 2). In addition, RNA sequences found only in one strain for each analysis with more than 100 rpm are also selected (Fig 2, categories 3 and 4 in right for each panel). Annotations of selected RNAs were analyzed by the mouse genome (GRCm39/mm39) and snoRNAs were picked up. Number of selected RNAs were summarized in Table 2. Notably, other RNAs included fragments of ribosomal RNAs (rRNAs), mitochondrial rRNAs, tRNA^Glu^, tRNA^Gly^ and long non-coding RNAs were also selected.

**Table 1:** Number of RNA sequences obtained by the NGS analysis.

Analysis	1		2		3	
Strain	B6	MSM	B6	MSM	B6	MSM
Read number	5,210,785	5,498,823	3,767,220	4,014,677	4,141,045	4,629,337
RNA sequences	180,679	190,655	319,431	359,410	381,742	406,614

**Table 2:** Number of selected RNAs.

Analysis	1		2		3	
Strain	B6	MSM	B6	MSM	B6	MSM
Box C/D snoRNA (SNORD)	15	15	19	18	16	14
Box H/ACA snoRNA (SNORA)	2	1	7	5	13	13
Others	7	10	6	9	10	13
Total	24	26	32	32	39	40

It is noted that several fragments with different 5′ and/or 3′ ends were found for each RNA. Sometimes, variations of the 3′ terminal residue were observed. These variations may have happened in the processing of snoRNA and/or degradation during the sample preparation. Thus, number of RNAs in Table 2 is smaller than number of selected sequences in Fig 2.

**Fig 3:**
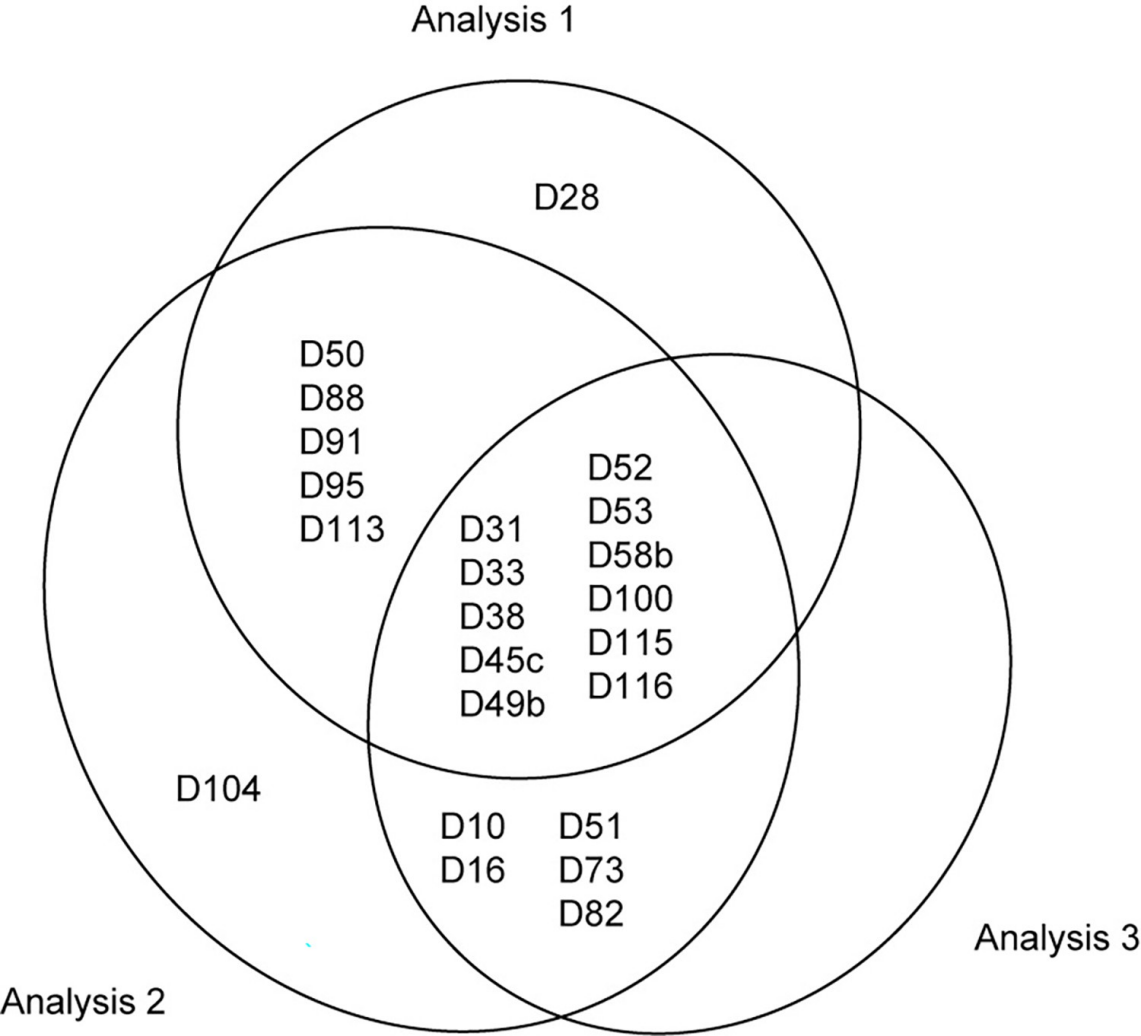
Differentially expressed SNORDs (D) detected in all the three analyses.

In the present study, we focused on SNORD which are most abundant small RNAs in cells. As shown in Table 2, 14 to 19 SNORDs were selected for each library. Twenty-three SNORDs were detected in total (Fig 3). Expression of SNORD28 was observed only in the analysis 1 and expression of SNORD104 was observed only in the analysis 2. In contrast, eleven SNORDs, SNORD31, SNORD33, SNORD38, SNORD45c, SNORD49b, SNORD52, SNORD53, SNORD58b, SNORD100, SNORD115 and SNORD116, were commonly selected among the three analyses and, thus, we further analyzed these 11 SNORDs. The sum of read numbers in rpm for each SNORD were shown in S1 Table and the target RNAs and host genes for these SNORDs are shown in S2 Table. Most of selected SNORDs target rRNAs except for SNORD115 and SNORD116 those targets are unknown. In contrast, host genes are varied.

### Analysis of the detected RNAs

**Fig 4:**
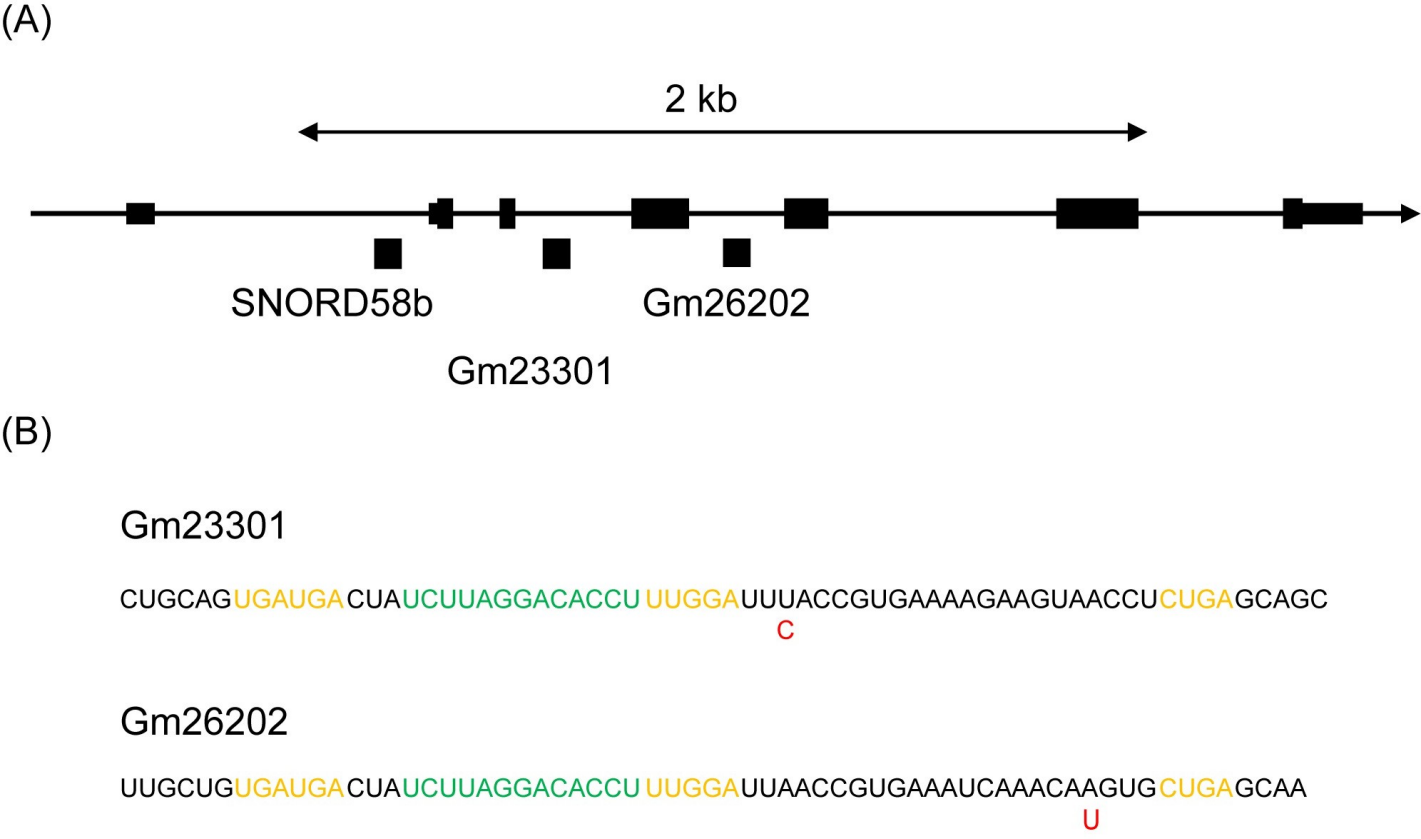
Examples of SNPs in snoRNAs. (A) Locus of Gm23301 and Gm26202 on the ribosomal protein L17 gene in Chromosome 18. Three snoRNAs are coded on the introns of this gene and two of them have SNPs between B6 and MSM. (B) SNPs in Gm23301 and Gm26202. Red: possible SNP residues. Orange: Box sequences. Green: Complementary sequence to target RNA.

Most of SNORDs could be paired between sequences specific for B6 and MSM (Table 3), indicating that the differences in read number are caused by SNPs rather than expression differences. SNORDs other than SNORD58b, SNORD115 and SNORD116 have single gene in the genome and one or two SNPs between B6 and MSM were found (Table 3, S3 Table). Small read numbers for the counter part of the strain pair are probably due to the contamination during the RNA and/or DNA library preparations. It is not the error in sequence analysis because any other sequences were merely detected. In the case of SNORD49b, two kinds of sequences are found for MSM with similar read numbers to each other. The reason of this phenomenon is unknown. Three genes of SNORD58b are coded in introns of the ribosomal protein L17 gene, and two of them have SNPs as shown in Fig 4. Among the three genes, products of Gm23301 and Gm26202 were identified in this study. Despite the expression of about 30,000 read number in both Gm23301 and Gm26202, SNORD58b has hardly been detected. SNORD58b may not be expressed in the brain of B6. Both Gm23301 and Gm26202 have SNPs as shown in Fig 4. Moreover, they target the same RNA.

**Table 3:** Read numbers of the detected box C/D snoRNAs (SNORDs).

		Total number of reads		
RNA	Sequence type	B6	MSM	SNP
SNORD31 (Gm23246)	B6	21,355	20	
	MSM	8	7,752	1
SNORD33	B6*^1^	9,463	10	
	MSM	0	12,135	1
SNORD38 (Gm22980)	B6	17,261	0	
	MSM	2	10,692	1
SNORD45c (Gm24494)	B6	25,907	19	
	MSM*^2^	39	33,028	1
SNORD49b	B6-1*^3^	6,951	63	
	MSM-1*^4^	33	5,347	2
	MSM-2	4	1,239	2
SNORD52	B6	13,247	19	
	MSM	11	11,995	1
SNORD53	MSM	0	8,990	1
SNORD58b (Gm23301)	B6-1*^2^	9,445	5	
	B6-2	22,196	8	
	MSM	16	21,664	1
SNORD58b (Gm26202)	B6	33,586	22	
	MSM	3	62,312	1
SNORD100	B6	4,345	0	
	MSM	0	5,793	1
SNORD115	B6-1	43,489	20	
	B6-2	2,750	19	
	B6-3	3,805	6	
	B6-4	21,571	0	
	B6-5	3,393	0	
	MSM-1	0	35,958	4
	MSM-2	3	4,438	1
SNORD116	B6-1	6,211	14	
	B6-2	4,486	26	
	B6-3	2,856	5	
	MSM	0	3,330	1

RNA sequences detected in all the three analyses are shown, if otherwise mentioned.

*^1^Detected only for analyses 2 and 3.

*^2^Detected only for analyses 1 and 2.

*^3^Detected only for analysis 1.

*^4^Detected only for analyses 1 and 3.

SNORD115 and SNORD116 are coded in introns of the small nucleolar RNA host gene 14 (Snhg14) as gene clusters [24]. Although these snoRNAs have sequence variations in each strain, differential expression is still observed by single SNPs (Table 3 and S3 Table).

### Analysis of SNPs

**Fig 5:**
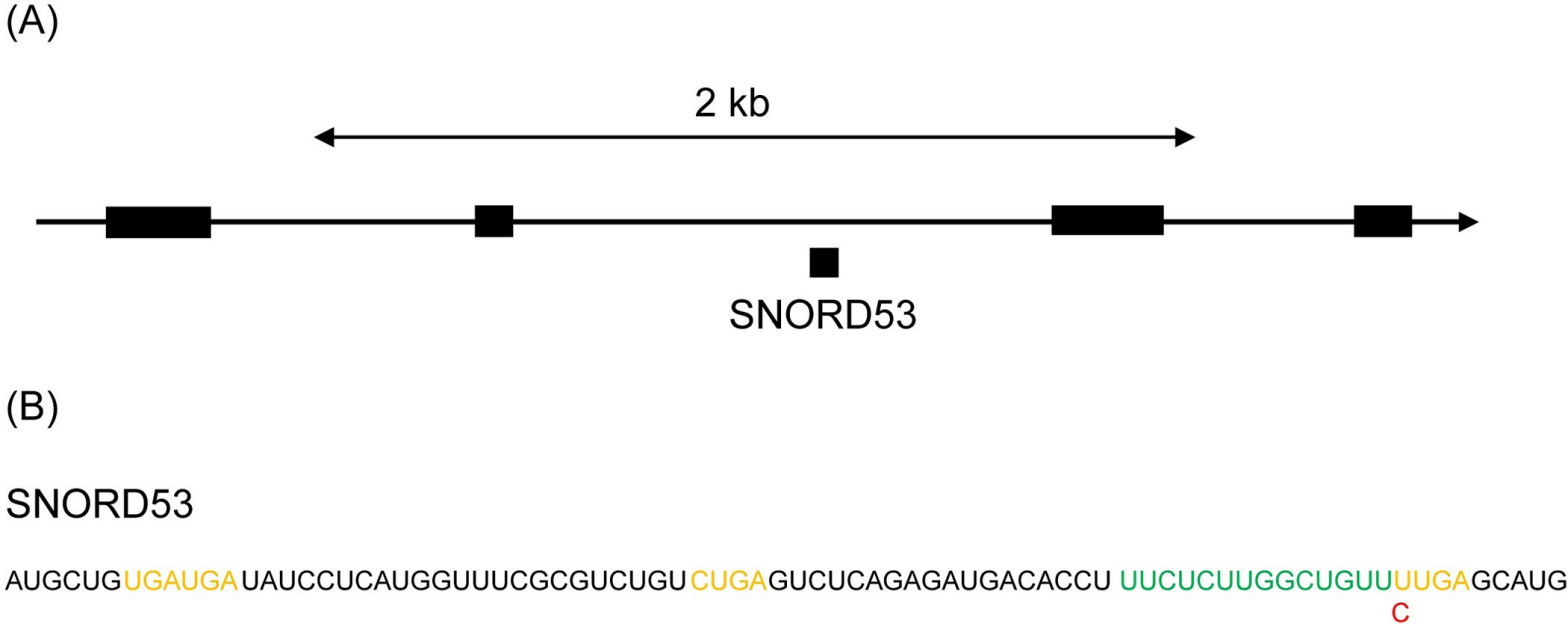
Examples of SNPs in snoRNA. (A) Locus of SNORD53 on the WD repeat domain 43 gene in chromosome 17. The snoRNA is coded on the introns of this gene. (B) SNPs in SNORD53. Red: possible SNP residues. Orange: Box sequences. Green: Complementary sequence to target RNA.

In the present study, most of SNPs were located neither on the box sequences nor complementary sequences for the target RNAs (S3 Table), suggesting that these SNPs are functionally silent. Only the exception is SNORD53, for which the SNP is in the first position of the box sequence CUGA (Fig 5). SNORD53 of MSM possesses the two consensus CUGA box sequences where that of B6 has a mutation in the second box according to the mouse genome (GRCm39/mm39). The target of SNORD53 is 28S rRNA and the second CUGA box sequence is conserved among mammalian. It is possible that SNORD53 lost its function in B6 and this may be a reason for loss of SNORD53 sequences in B6. The expression of the mutated SNORD53 may be suppressed and/or dysfunctional SNORD53 may be degraded in B6.

## Discussion

In this study, differential expressions between two mouse strains, B6 and MSM, of box C/D snoRNA were analyzed by the large-scale sequence analysis. In most cases, differential expressions were found to be due to SNPs rather than different regulation of gene expression. In most cases, no SNPs were found in functional sequences such as the box sequences or the complementary sequences to target RNAs. As described below, SNORD53 was identified only for MSM, indicating that SNP-based analysis is effective for the analysis of differential expressions between strains. Thus, it was demonstrated that the comparison between the two strains give new insight for the gene expression regulation. It should be noted that SNORD115 and SNORD116, which form large gene clusters, are also basically distinguishable by SNPs despite the polymorphisms in these loci. For SNORD115 as example, B6-1 to 5 are B6 specific and MSM-1 and 2 are MSM specific as shown in Table 3. It is noted that sequences shared between the two strains were not detected in this analysis. Functionally silent SNPs can be used for the discrimination of expressed alleles. Similar results were also obtained for SNORD116. There is a possibility that it can be used for functional analysis of SNORD115 and SNORD116.

Expression of SNORD53 may be different between the two strains; this snoRNA was found only for MSM. In fact, the sequence of SNORD53 was extracted from the three data for B6 to find negligible read numbers (3, 20 and 14 rpm for each trial). Because SNORD53 in B6 has a mutation in the box sequences and this may cause the gene silencing or degradation of the product. Thus, it was demonstrated that the comparison between the two strains give new insight for the gene expression regulation. Because the genome sequence of MSM has been determined, it is possible to identify snoRNAs with SNPs. However, the method in this study can show the existence of SNPs and expression levels simultaneously. In addition, this method can be applied to strains for which the genome sequence is not determined. Further analysis will give more information on the differential expression of small RNAs.

As shown in Fig 3, more than half snoRNAs were shared among the three results despite of the different DNA library kits. In the analysis 1 (TruSeq), 5′ and 3′ adaptors were simply ligated and amplified by PCR. In the analysis 2 (NEBNext), the primers for reverse transcription were added after the 3′ adaptor ligation to prevent the formation of adaptor dimer. Then the 5′ adaptor was ligated and amplified. In contrast, in the analysis 3 (SMARTer), RNAs were circularized after 3′ adaptor ligation and, then, reverse transcribed and amplified. SNORDs seem to be effectively incorporated to the DNA libraries by the three methods. It is noted that the sequences of snoRNAs obtained for C57BL/6J were completely agree with those of GRCm39/mm39, suggesting the accuracy of the sequencing as well as the suitability of C57BL/6J as a control subspecies for this work.

Some snoRNAs are located at the imprinted loci and are affected by the expression mechanism of the imprinted gene [18]. It is known that snoRNAs are incorporated into the gene expression regulation because the expression disruption of the imprinted locus affects the expression of snoRNA groups located there and causes abnormal development and diseases [23].

All mammals, including human, inherit a set of chromosomes from father and mother, and both parents-derived genes are expressed [15]. However, for some genes, the gene expression mechanism called genomic imprinting is known, where only one allele of the two is expressed in a parental-of-origin manner. [25, 26]. Most genes that is subject to the genomic imprinting control have important roles in regulating the development of individuals and the activities of living organisms [27]. It has been demonstrated that monoallelic expression of RNAs can be analyzed by using the F1 hybrid mice between B6 and MSM [28]. In the hybrid F1, there are several genes that show different expression patterns compared to fathers and mothers. For example, incompatibility due to strain difference occurs due to factors such as mismatching combinations of transcription regulatory factors [28]. To analyze the incompatibility, it is important to know the normal expression pattern and the proposed method based on the SNPs are useful for such analyses. In addition, the proposed experimental system using SNPs can be used as a tool for analyzing imprinting by providing the reference data for the expressions.

## Supporting information

S1 Table Read numbers of selected box C/D snoRNAs (SNORDs) in rpm. (PDF)

S2 Table Properties of the selected box C/D snoRNAs (SNORDs). (PDF)

S3 Table SNPs on the detected snoRNAs. (PDF)
