# Supplementary material for: Expression analysis of box C/D snoRNAs with SNPs between C57BL/6 and MSM/Ms strains in male mouse
Source: PLoS One. 2023 Jul 10;18(7):e0288362. doi: 10.1371/journal.pone.0288362 (PMC10332580; doi:10.1371/journal.pone.0288362)
Supplement: S1 Table — (PDF) [file pone.0288362.s001.pdf]

Saito et al., Expression analysis of Box C/D snoRNAs with SNPs between C57BL/6 and MSM/Ms strains in mouse

Supplementary Table 1. Read numbers of selected Box C/D snoRNAs (SNORDs) in rpm.

Strain	B6			MSM			Total
Sample	1	2	3	1	2	3	
SNORD58	5248.9	6773.7	4456.4	9524.8	5958.1	3653.2	35615.1
SNORD115	3110.5	6524.9	13063.4	3950.5	3062.5	4886.5	34598.2
SNORD116	4131.3	6575.1	782.4	4152.3	3399.5	706.6	19747.2
SNORD45c	1752.3	5395.5	103.6	2718.4	5028.1	0.0	14997.9
SNORD31	953.0	3480.9	1073.9	3309.4	3822.7	625.2	13265.1
SNORD38	850.5	1766.0	1491.9	590.1	1042.2	904.6	6645.3
SNORD52	433.7	1847.8	974.9	353.3	1924.9	506.1	6040.7
SNORD33	0.0	139.1	2158.6	268.2	117.3	2203.1	4886.4
SNORD49b	1335.9	0.5	832.4	245.3	80.0	1161.5	3655.6
SNORD100	0.0	825.5	298.2	224.4	559.4	499.6	2407.1
SNORD53	0.0	0.0	0.0	592.9	946.8	416.7	1956.3
