# Supplementary material for: Expression analysis of box C/D snoRNAs with SNPs between C57BL/6 and MSM/Ms strains in male mouse
Source: PLoS One. 2023 Jul 10;18(7):e0288362. doi: 10.1371/journal.pone.0288362 (PMC10332580; doi:10.1371/journal.pone.0288362)
Supplement: S2 Table — (PDF) [file pone.0288362.s002.pdf]

Saito et al., Expression analysis of Box C/D snoRNAs with SNPs between C57BL/6 and MSM/Ms strains in mouse

Supplementary Table 2. Properties of the selected Box C/D snoRNAs (SNORDs).

No.	snoRNA name	Target RNA	Chr.	Host gene
1	SNORD31	28S rRNA	19	small nucleolar RNA host gene 1 (Snhg1)
2	SNORD33	18S rRNA	7	ribosomal protein L13A (Rpl13a)
3	SNORD38	28S rRNA	4	ribosomal protein S8 (Rps8)
4	SNORD45c	18S rRNA	3	Rab geranylgeranyl transferase, b subunit (Rabggtb)
5	SNORD49b	28S rRNA	11	long non-coding RNA
6	SNORD52	28S rRNA	17	long non-coding RNA
7	SNORD53	28S rRNA	17	WD repeat-containing protein 43 (Wdr43)
8	SNORD58b	28S rRNA	18	ribosomal protein L17 (Rpl17)
9	SNORD100	18S rRNA	10	ribosomal protein S12 (Rps12)
10	SNORD115	Unknown	7	small nucleolar RNA host gene 14 (Snhg14)
11	SNORD116	Unknown	7	small nucleolar RNA host gene 14 (Snhg14)

No.	Representative sequence
1	CACCCUGAUGAACUGAAUACCGCCCCAGUCUGAUAGCUGUGGAGAAAGGUAUUUUCUGAGU
2	AGCUUGUGAUGAGACAUCUCCCACUCAUGUUCGAGUUGCUCGACUAUGAGAUGACUCUACAUGCACUACCAUCUGAGGCUG
3	CUCGGUGAUGAGAACUUUGUCCAGUUCUGCUGCUGAUCUCUUAAGUGAGGAUGAAGUUAUCUGAGG
4	GGUCAAUGAUGUGUUGGCAUGUAUUAUCUGAAUUCGCUGAUGUGUCAUAACACUUUAGCUCUAGAAUUACGCUGAGACCU
5	UGCAAUGAUGAUGAAACUAGAAAAAAAAGGAAGUGCCGUCCGAUGCGACAACUGACGACAUCCCUAGUUAGCUGACU
6	UGAGAGUGAUGAUUUCACAGACUAGAGUCUCUGACGCUGUCCUUGAUGUCAGCUAUAAAUCUGACUCA
7	AUGCUGUGAUGAUAUCCUCAUGGUUUCGCGUCUGUCUGAGUCUCAGAGAUGACACCUUUCUCUUGGCUGUUUGAGCAUG
8	UUGCUGUGAUGACUAUCUUAGGACACCUUUGGAUUAACCGUGAAAUCAAACAAGUGCUGAGCAA
9	GUACAUGAUGAAAACAGUCUCCCUCUUCUGAAUCUCGCUGAGGAAACUGCACGUCACCCUCCUGAAA
10	GGUCAAUGAUGACAACCCAAUGUCAUGAACAAAGGUGAUGACAUAAAAUUCAUGCUCAAUAGGAUUACGCUGAGGCCC
11	GAUCUAUGAUGAUUGCCAGUCAAACAUUCCUUGGAAAAGCUGAACAAAAUGAGUGAAAACUCUGUACCGCCACUCUCAUCGGAACUGAGGUCC
