# Supplementary material for: Expression analysis of box C/D snoRNAs with SNPs between C57BL/6 and MSM/Ms strains in male mouse
Source: PLoS One. 2023 Jul 10;18(7):e0288362. doi: 10.1371/journal.pone.0288362 (PMC10332580; doi:10.1371/journal.pone.0288362)
Supplement: S3 Table — (PDF) [file pone.0288362.s003.pdf]

Supplementary Table 3. SNPs on the delected snoRNAs (SNORDs).

Red: possible SNP residues. Orange: Box sequences. Green: complementary sequence to target RNA. Blue: genetic varieties in SNORD115 and 116.

RNA	Strain	Sequence
SNORD31 (Gm23246)	B6	CACCCUGAUGAACUGAAUACCGCCCCAGUCUGAUAGCUGUGGAGAAAGGUAUUUUCUGAGU
	MSM	CACCCUGAUGAACUGAAUACCGCCCCAGUCUGAUAGCUGUGAAGAAAGGUAUUUUCUGAGU
SNORD33	B6	AGCUUGUGAUGAGACAUCUCCCACUCAUGUUCGAGUUGCUCGACUAUGAGAUGACUCUACAUGCACUACCAUCUGAGGCUG
	MSM	AGCUUGUGAUGAGACCUCUCCCACUCAUGUUCGAGUUGCUCGACUAUGAGAUGACUCUACAUGCACUACCAUCUGAGGCUG
SNORD38 (Gm22980)	B6	CUCGGUGAUGAGAACUUUGUCCAGUUCUGCUGCUGAUCUCUUAAGUGAGGAUGAAGUUAUCUGAGG
	MSM	CUCGGUGAUGAGAACUUUGUCCAGUUCUGCUGCUGAUCUCU_AAGUGAGGAUGAAGUUAUCUGAGGA
SNORD45c (Gm24494)	B6	GGUCAAUGAUGUGUUGGCAUGUAUUAUCUGAAUUCGCUGAUGUGUCAUAACACUUUAGCUCUAGAAUUACGCUGAGACCU
	MSM	GGUCAAUGAUGUGUUGGCAUGUAUUAUCUGAACUCGCUGAUGUGUCAUAACACUUUAGCUCUAGAAUUACGCUGAGACC
SNORD49b	B6	UGCAAUGAUGAUGAAACUAG__AAAAAAAAGGAAGUGCCGUCCGAUGCGACAACUGACGACAUCCCUAGUUAGCUGACU
	MSM-1	UGCAAUGAUGAUGAAACUAGAAAAAAAAAGGAAGUGCCGUCCGAUGCGACAACUGACGACAUCCCUAGUUAGCUGACU
	MSM-2	UGCAAUGAUGAUGAAACUAAA_AAAAAAAAGGAAGUGCCGUCCGAUGCGACAACUGACGACAUCCCUAGUUAGCUGACU
SNORD52	B6	UGAGAGUGAUGAUUUCACAGACUAGAGUCUCUGACGCUGUCCUUGAUGUCAGCUAUAAAUCUGACUCA
	MSM	UGAGAGUGAUGAUUUCACAGACUAGAGUCUCUGACACUGUCCUUGAUGUCAGCUAUAAAUCUGACUCA
SNORD53	MSM	AUGCUGUGAUGAUAUCCUCAUGGUUUCGCGUCUGUCUGAGUCUCAGAGAUGACACCUUUCUCUUGGCUGUCUGAGCAUG
SNORD58b (Gm23301)	B6	CUGCAGUGAUGACUAUCUUAGGACACCUUUGGAUUUACCGUGAAAAGAAGUAACCUCUGAGCAGC
	MSM	CUGCAGUGAUGACUAUCUUAGGACACCUUUGGAUUCACCGUGAAAAGAAGUAACCUCUGAGCAGC
SNORD58b (Gm26202)	B6	UUGCUGUGAUGACUAUCUUAGGACACCUUUGGAUUAACCGUGAAAUCAAACAAGUGCUGAGCAA
	MSM	UUGCUGUGAUGACUAUCUUAGGACACCUUUGGAUUAACCGUGAAAUCAAACAUGUGCUGAGCAA
SNORD100	B6	GUACAUGAUGAAAACAGUCUCCCUCUUCUGAAUCUCGCUGAGGAAACUGCACGUCACCCUCCUGAAA
	MSM	GUACAUGAUGAAAACAGUCUCCCUCUUCUGAACCUCGCUGAGGAGACUGCACGUCACCCUCCUGAAA
SNORD115	B6-1	GGUCAAUGAUGACAACCCAAUGUCAUGAACAAAGGUGAUGACAUAAAAUUCAUGCUCAAUAGGAUUACGCUGAGGCCC
	B6-2	GGUCAAUGAUGACAACCCAAUGUCAUGAAGAAAAGUGAUGACAUAAAAUUCAUGCUCAAUAGGAUUACGCUGAGGCCC
	B6-3	GGUCAAUGAUGACAACCCAAUGUCAUGAAGAACGGUGAUGACAUAAAAUUCAUGCUCAAUAGGAUUACGCUGAGGCCC
	B6-4	_GUCAAUGAUGAAAACCCAAUGUCAUGAAGAAAAGUGAUGACAUAAAAUUCAUGCUCAAUAGGAUUACGCUGAGGCC
	B6-5	_GUCAAUGAUGAAAACCCAAUGUCAUGAAGAAAAGUGAUGACAUAAAAUUCAUGCUUAAUAGGAUUACGCUGAGGCC
	MSM-1	GGUCAGUGAUGACAACCCAAUGUCAUGAAUAAACGUGAUGACAUAAAAUUCAUGCUCAAUAGGAUUACUCUGAGGCCC
	MSM-2	GGUCAAUGAUGACAACCGAAUGUCAUGAAGAAAGGUGAUGACAUAAAAUUCAUGCUCAAUAGGAUUACGCUGAGGCC
SNORD116	B6-1	GAUCUAUGAUGAUUGCCAGUCAAACAUUCCUUGGAAAAGCUGAACAAAAUGAGUGAAAACUCUGUACCGCCACUCUCAUCGGAACUGAGGUCC
	B6-2	GAUCUAUGAUGAUUCCCAGUCAAACAUUCCUUGGAAAAGCUGAACAAAAUGAGUGAAAACUCUGUACCGUCACUCUCAUCGGAACUGAGGUCC
	B6-3	_AUCUAUGAUGAUUCCCAGUCAAACAUUCCUUGGAAAAGCUGAACAAAAUGAGUGAAAACUCUGUACCGCCACUCUCAUCGGAACUGAGU
	MSM	_AUCUAUGAUGAUUUCCAGUCAAACAUUCCUUGGAAAAGCUGACCAAAAUGAGUGAAAACUCUGUACCACCACUCUCAUCGGAACUGAGG
